# Editorial: Insights in Inflammation Pharmacology: 2021

**DOI:** 10.3389/fphar.2022.928535

**Published:** 2022-05-27

**Authors:** Paola Patrignani, Dieter Steinhilber

**Affiliations:** ^1^ Department of Neuroscience, Imaging and Clinical Science, and CAST, “G. d’Annunzio” University, Chieti, Italy; ^2^ Institut fuer Pharmazeutische Chemie, Universitaet Frankfurt, Frankfurt am Main, Germany

**Keywords:** inflammatioin, resolution, fibrosis, anaphyalaxis, 5-lipoxygenase, cyclooxygenase-2

Inflammation is a complex physiological response to offending agents such as viruses, bacteria, toxic chemicals, or an injury, which activates many cell types, including immune, stromal, vascular cells, and platelets, to start the healing process, which involves the induction of a plethora of healing signaling pathways. However, these defense reactions result in the development of pain, swelling, bruising, or redness. Whether the inflammatory process persists and leads to a chronic state that can cause severe damage to many tissues and is associated with developing serious diseases such as tissue fibrosis, atherosclerosis, and cancer (Dovizio et al., 2017; Ballerini et al., 2022), depends on the ability or failure to induce resolution of the inflammatory process.

Despite the availability of therapeutics to affect symptoms of inflammation and constrain cellular or molecular players involved in chronic inflammation, novel mechanistic knowledge on the resolution of inflammation can translate into more specific treatments based on individual phenotypes, thus improving the efficacy and safety of drugs.

Insights in inflammation pharmacology 2021 Research Topic examines unresolved issues in these settings and combines: 1) a review by Wu describes the role of 5-methoxytryptophan (5-MTP) synthesis in fibrosis and its potential as a lead compound for new anti-fibrotic drugs; 2) a study by Cho et al. explores the benefits for the control of anaphylaxis with increased dimerized translationally controlled tumor protein (dTCTP) by dTCTP-binding peptide 2 (dTBP2); 3) a review by Hong et al. on CC chemokine receptor 7 (CCR7) as a potential target of immunotherapy; 4) a paper by Golenkina et al. describes novel mechanisms of leukotriene(LT)B_4_ synthesis regulation; 5) Kahnt et al. report novel off-target effects of available 5-lipoxygenase (LOX) inhibitors; 6) Stamatakis et al. show the effect of COX-2 and its effector genes in a mouse model of colorectal cancer and metastasis.

Fibrosis is a shared biological event in many forms of organ failure, thus leading to morbidity and mortality worldwide. Most chronic inflammatory diseases are associated with fibrosis characterized by aberrant fibroblast activation leading to the accumulation of excess extracellular matrix components and inadequate tissue repair. Macrophages can accumulate in the microenvironment and promote fibroblast activation, but also they can transdifferentiate to myofibroblasts through the phenomenon called macrophage myofibroblast transition (MMT) (Murray et al., 2011). Wu has described a possible strategy to prevent tissue fibrosis via 5-methoxytryptophan (5-MTP), which inhibits macrophage activation and blocks fibroblast (including hepatic stellate cell) differentiation to myofibroblasts. The endogenous 5-MTP derived from tryptophan catabolism via the tryptophan hydroxylase pathway represents a lead compound for developing new anti-fibrotic drugs. These findings are relevant considering that therapeutic strategies to prevent fibrosis are unavailable.

In many inflammatory settings, including host defense against parasitic infection and allergic reactions, mast cells, long-lived tissue-resident cells, play an essential role (Galli et al., 2008). Mast cells release a wide variety of inflammatory mediators. In anaphylaxis, these events culminate in serious respiratory, cardiovascular, and mucocutaneous manifestations that can be fatal. Despite effective treatments in this setting being available, such as adrenaline as a first-line treatment, glucagon as a second-line treatment, and anti-histamines as a third-line treatment, there are still unmet therapeutic needs for anaphylaxis (Tanno et al., 2019). A better understanding of the mechanisms and causes of anaphylaxis will help develop therapeutics based on precision medicine. Dimerized translationally controlled tumor protein (dTCTP) has been proposed as a pharmacological target for allergic diseases. In particular, dTCTP is involved in mast cell histamine release. dTCTP-binding peptide 2 (dTBP2) has been reported to attenuate severe allergic rhinitis and asthma. Cho et al. showed that *in vitro* dTBP2 suppressed mast cell degranulation and histamine release, and the *in vivo* administration was beneficial for the control of anaphylaxis associated with increased dTCTP.

Dendritic cells (DCs) are involved in innate and acquired immunity, and their migration is strictly relevant to many immune-related diseases, such as dermatomyositis, atherosclerosis, and viral infections (Liu et al., 2021). The 7-fold transmembrane G protein-coupled receptor (GPCR) CC chemokine receptor 7 (CCR7) is expressed in antigen-carrying mature DCs. CCR7 mediates the migration of conventional DCs (cDCs) and the trafficking of plasmacytoid DCs (pDCs) to draining lymph nodes (dLNs). Hong et al. extensively discussed the regulatory network of CCR7 signaling, examining possible strategies to weaken or reinforce DC migration. They show that targeting the CCR7 network can be an effective clinical therapy.

5-Lipoxygenase (5-LOX) is the crucial enzyme in the formation of pro-inflammatory leukotrienes (LT) from arachidonic acid (AA), which play an important role in several inflammatory diseases. Several pathogens have been shown to activate 5-LOX, and the resulting synthesis of leukotrienes is critical for host survival. In particular, leukotriene B_4_ (LTB_4_) is a neutrophil chemoattractant important in neutrophil swarming. This phenomenon significantly boosts the accumulation of neutrophils at sites of injury or infection and serves to engulf microbes and their clusters that are too large for individual neutrophils to kill. Golenkina et al. reported that preincubation of human neutrophils with *Salmonella typhimurium* strongly stimulated LTB_4_ production induced by the bacterial chemoattractant, peptide *N*-formyl-L-methionyl-L-leucyl-l-phenylalanine (fMLF), thus promoting neutrophil swarming and elimination of pathogens simultaneously.

The 5-LOX activation is involved in many disorders associated with inflammation. Thus, 5-LOX inhibitors (including AA-861, BWA4C, C06, CJ-13,610, zileuton, and pan-LO inhibitor nordihydroguaiaretic acid) have been tested in many preclinical and clinical studies. However, heterogeneity in their efficacy has been detected, and it has been suggested that besides the inhibition of LT biosynthesis, these compounds may have additional off-target effects. Kahnt et al. showed that 5-LOX inhibitors act as potent inhibitors of PGE_2_ export from cells. In addition, 5-LO inhibitors may serve as new scaffolds for developing potent prostaglandin export inhibitors.

Cyclooxygenase-2 (COX-2) is highly induced in inflammation-driven diseases such as colorectal cancer (CRC) (Ballerini et al., 2022). Stamatakis et al. showed that many genes expressed in the mouse colorectal cancer cell line CT26 are COX-2-regulated. Interestingly, they show that the analysis of the expression profile of COX-2-effector genes in Ficoll (Blood-Nucleated Cells) or Parsortix (an isolation system for circulating tumor cells, CTCs) may offer valuable diagnostic and prognostic information. These findings should be confirmed in further studies.
